# Supplementary material for: Interferon lambda 4 impairs hepatitis C viral antigen presentation and attenuates T cell responses
Source: Nat Commun. 2021 Aug 12;12:4882. doi: 10.1038/s41467-021-25218-x (PMC8360984; doi:10.1038/s41467-021-25218-x)
Supplement: Supplementary file 1 — Supplementary Information [file 41467_2021_25218_MOESM1_ESM.pdf]

Supplementary Information

Interferon lambda 4 impairs hepatitis C viral antigen presentation and attenuates T cell responses

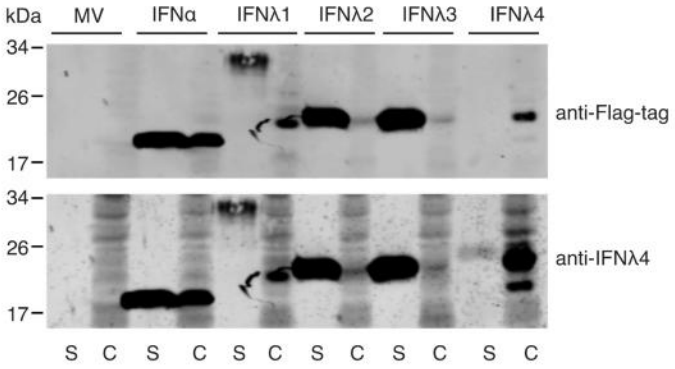

Supplementary Figure 1. IFNλ4 is poorly secreted. Huh7 cells were transfected with Flag-tagged expression vectors for IFNα and IFNλ1-4 or a mock vector (MV) and cell lysates (C) and supernatants (S) were harvested 48 hours post-transfection. IFN proteins were detected by Flag-tag specific western blotting (upper panel). Without stripping, membranes were re-probed with an IFNλ4-specific antibody (bottom panel). kDa, kilodalton. Data are representative of n=3 independent experiments.

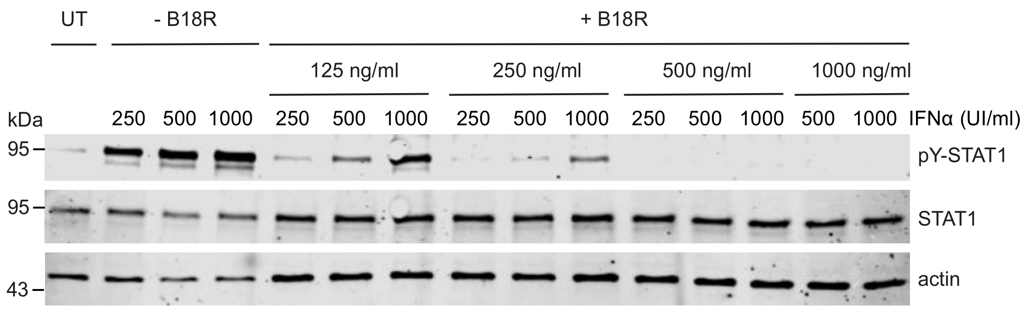

Supplementary Figure 2. B18R prevents IFNα-mediated STAT1 activation. Huh7-LR cells were pre-incubated with the type I IFN inhibitor B18R at 125, 250, 500, 1000 ng/ml for 2 hours. After washing, the cells were incubated with recombinant IFNα (Roferon-A) at 250, 500, 1000 UI/ml for 30 minutes or left untreated (UT). STAT1 activation (pY-STAT1, STAT1) was assessed by Western blotting. kDa, kilodalton. Data are representative of n=3 independent experiments.

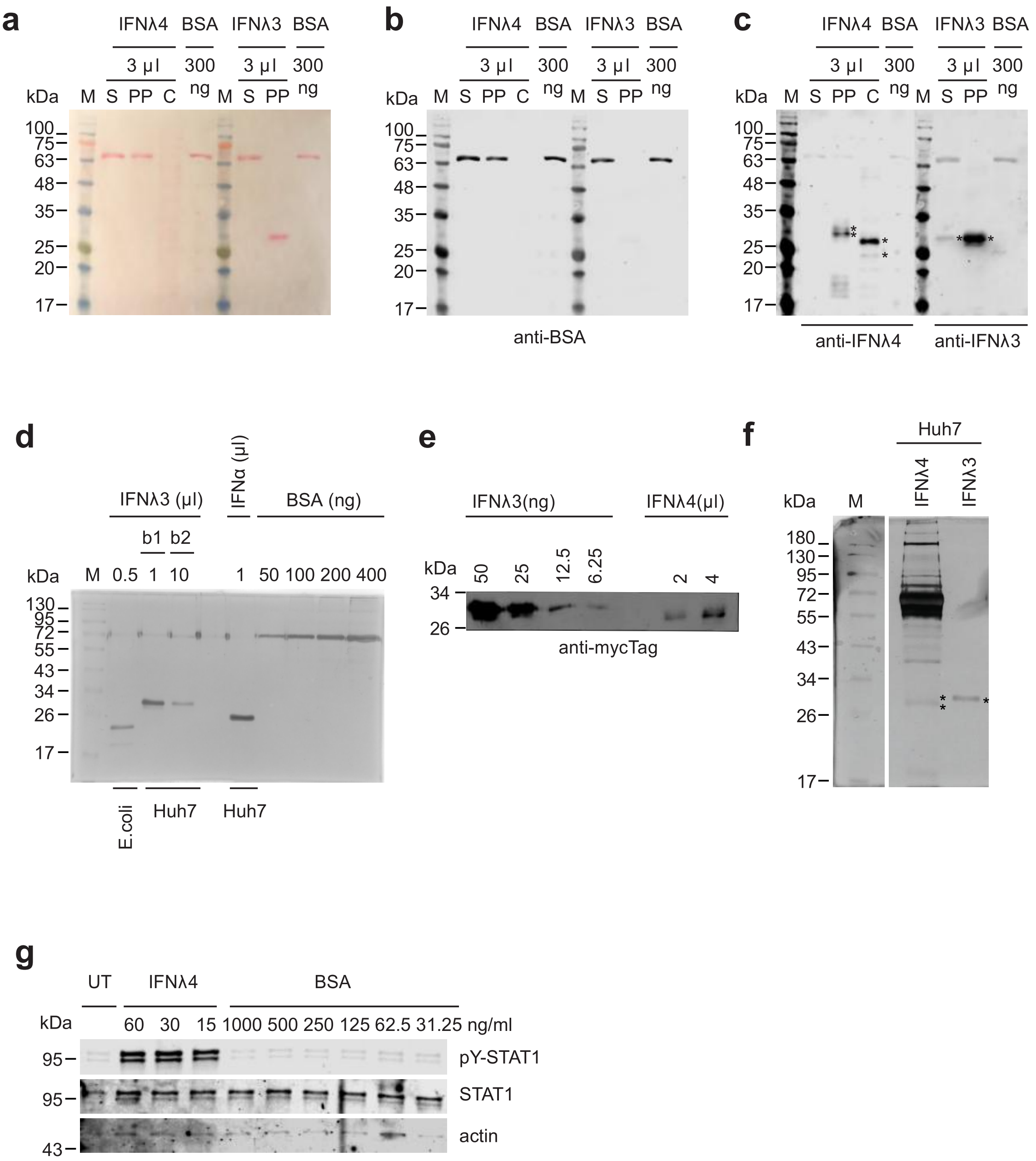

Supplementary Figure 3. Purification and quantification of IFNλ3 and IFNλ4. Huh7 cells were transfected with Myc-His-tagged expression vectors for IFNα, IFNλ4 and IFNλ3. 48 hours later, recombinant IFNα, IFNλ4 and IFNλ3 were purified from the culture supernatant by Ni affinity chromatography, concentrated and dialyzed against PBS as described in the Methods. **(a-c)** IFNλ4 and IFNλ3 in the input cell culture supernatants (S) and in the purified preparations (PP) were analyzed by Western blotting. Intracellular (C) IFNλ4 was included for comparison. BSA (300ng) served as a protein reference. Data are representative of n=3 independent experiments. **(a)** Visualization of membrane bound proteins by Ponceau staining after protein transfer.

(b) The same membrane was then probed with a BSA specific antibody. **(c)** Without stripping, the membrane was then probed with IFNλ4 or IFNλ3 specific antibodies, respectively. IFNλ4 and IFNλ3 proteins are indicated by asterisks. Secreted IFNλ4 is glycosylated and has an apparent MW of 28-30 kDa. Bands smaller than 20 kDa represent non-functional degradation products of IFNλ4. Intracellular IFNλ4 exists in two isoforms, a partially glycosylated form of ~ 27 kDa and a non-glycosylated form of ~ 24 kDa. As expected, secreted IFNλ3 appears as a single band of ~ 26 kDa. **(d)** SDS-PAGE separation and silver staining of purified IFNλ3 from E. coli and of two batches (b1 and b2) of Huh7 cell culture supernatant, and IFNα purified from Huh7 cell culture supernatant. Concentrations were determined using bovine serum albumin (BSA) as a standard. Purified IFNα was not used any further. **(e)** IFNλ4 purified from Huh7 cell culture supernatant was quantified against the purified IFNλ3 (b1) shown in (d). **(f)** 50 ng of purified IFNλ4 and IFNλ3 were visualized by silver staining following SDS-PAGE. **(g)** Huh7-LR cells were stimulated with purified IFNλ4 or BSA at the indicated concentrations for 30 minutes or left untreated (UT). STAT1 activation was assessed as described in Fig.2. M, protein molecular weight marker; kDa, kilodalton.

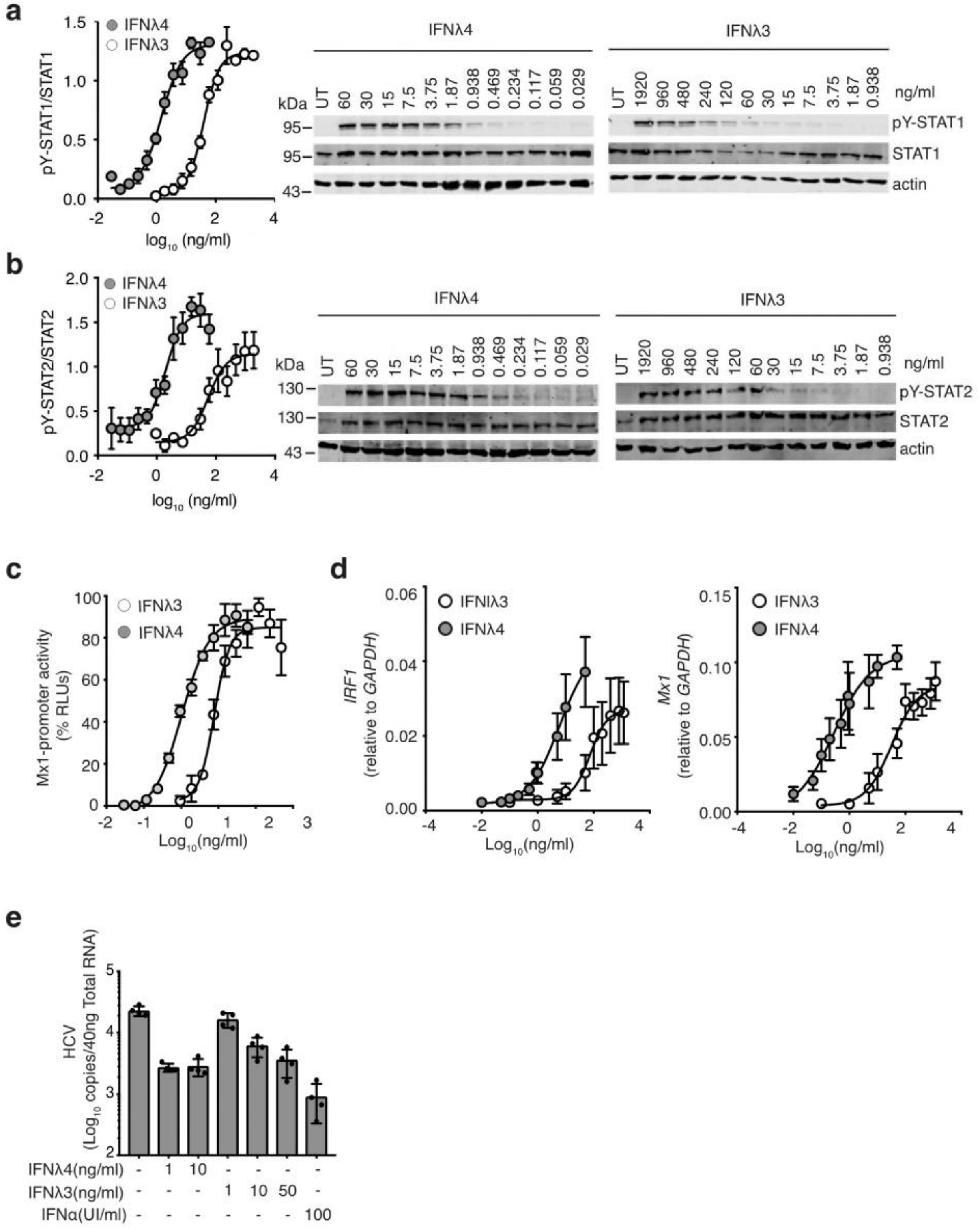

Supplementary Figure 4. Relative IFNλ4 and IFNλ3 activity. (a-b) Huh7-LR cells were stimulated with purified IFNλ4 or IFNλ3 at the indicated concentrations for 30 minutes or left untreated (UT). STAT1 **(a)** and STAT2 **(b)** activation was assessed by Western blotting as described in Fig. 2. The Western blot signals were quantified as described in Methods and the ratios of phospho-STAT1/2 to total STAT1/2 were plotted

as a dose-response curve. Results are mean±SEM from n=3 independent experiments. **(c)** Huh7-LR cells were transfected with an ISRE-Mx1 firefly luciferase reporter construct pGL3-Mx1P-FF-Luc and 18 hours later treated with serial dilutions of IFNλ4 or IFNλ3. ISRE promoter activation was evaluated 6 hours later by luciferase reporter gene expression. Relative luminescence units as percentage (% RLU) were shown. **(d)** Huh7-LR cells were stimulated with serial dilutions of IFNλ4 or IFNλ3 for 6 hours. Transcriptional induction of ISGs (*IRF1*, *Mx1*) was then quantified in total cellular RNA by RT-qPCR. mRNA expression was calculated relative to that of the house keeping gene *GAPDH*. Filled circles, IFNλ4; empty circles, IFNλ3; kDa, kilodalton. **(e)** Huh7.5.1-LR1 cells were infected for 6 hours with cell-culture derived HCV (JFH1-D183) at MOI=1. Cells were then left untreated or were treated with purified IFNλ4 (1 or 10 ng/mL) or IFNλ3 (1, 10 or 50 ng/mL) or IFNα (100 IU/mL) for 48 hours. HCV replication was monitored by HCV-specific RT-QPCR using total cellular RNA. c-e, Results are mean±SEM from n=4 biological replicates.

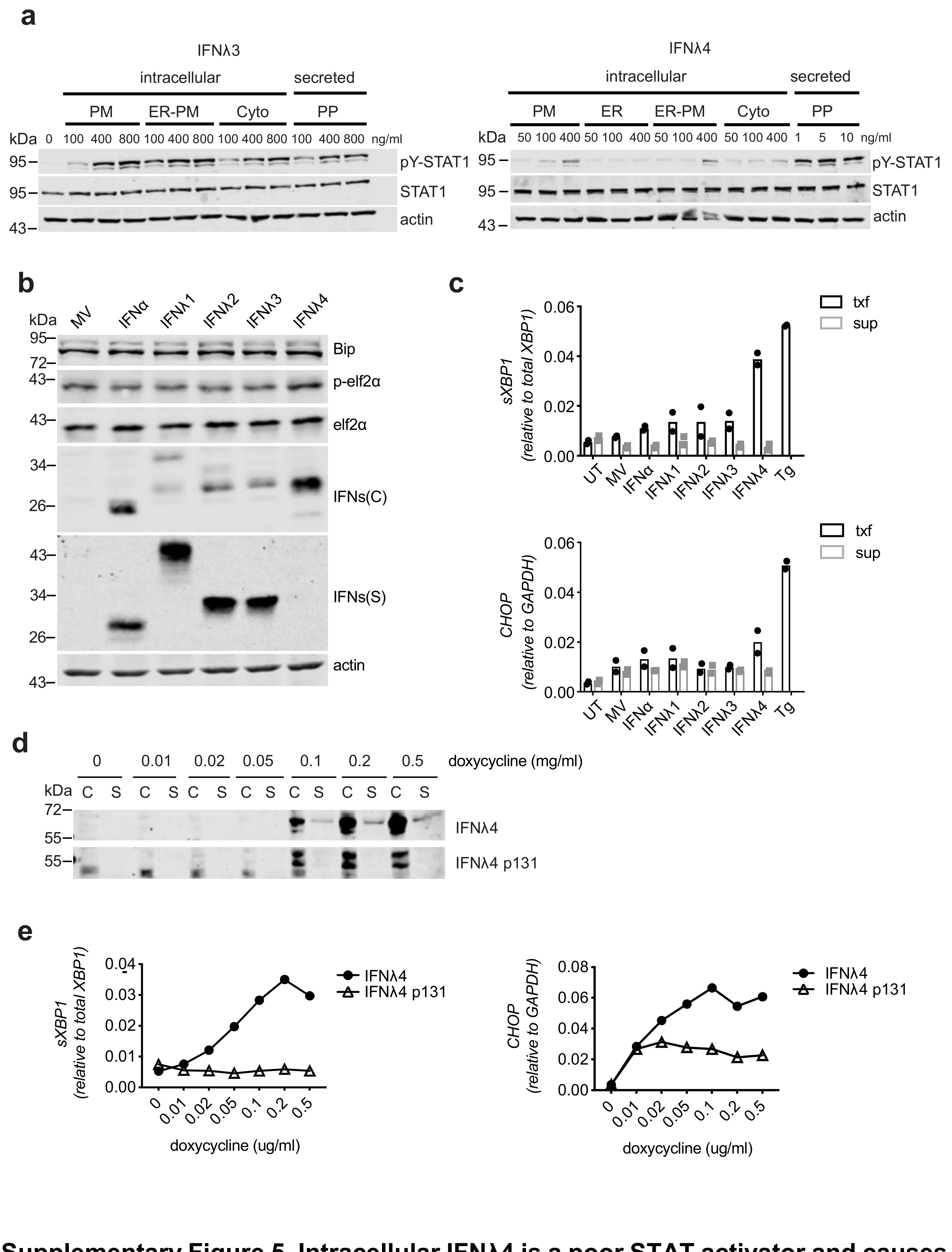

Supplementary Figure 5. Intracellular IFNλ4 is a poor STAT activator and causes ER stress. (a) Intracellular IFNλ4 is a poor STAT-activator. Huh7-LR cells were treated with the indicating amounts of IFNλ3 (left panel) or IFNλ4 (right panel) present in the subcellular fractions shown in Fig. 4 or with purified (PP) IFNλ4 or IFNλ3 for 30 minutes. Stimulated Huh7-LR cells were lysed and analyzed for pY-STAT1, STAT1 and actin as loading control by western blotting. Data are representative of n=3

independent experiments. **(b,c)** Intracellular IFNλ4 induces ER stress. **(b)** Huh7 cells were transfected with Myc-His-tagged IFN expression vectors or a mock vector (MV). Cell lysates and cell culture supernatants were collected 24 hours after transfection. Cell lysates were analyzed by Western blotting for the ER stress markers Bip and phospho-eIF2α and for total eIF2α and intracellular IFNs (IFNs(C)) using marker-specific antibodies and an anti-Myc-tag antibody, respectively. IFN proteins in the supernatants (IFNs(S)) were also detected with an anti-Myc-tag antibody. Data are representative of n=4 independent experiments. **(c)** Huh7 cells were transfected with IFN expression vectors (txf, black) or treated with IFN containing supernatants of IFN-expression vector transfected Huh7 cells (sup, grey). Total cellular RNA was extracted 24 hours later and the mRNAs of the ER stress markers *sXBP1* (upper panel) and *CHOP* (lower panel) were analyzed as described in Fig. 4d. Results are presented as mean of n=2 biological replicates. UT, untransfected or untreated; MV, mock vector transfected; Tg, Tapsigargin. **(d)** Expression of the wild-type IFNλ4-GFP fusion protein or the non-functional mutant IFNλ4 p131-GFP was induced by increasing amounts of doxycycline in inducible HepG2 cell system (a gift from Dr. Prokunina-Olsson) for 24 hours. Intracellular (C) and secreted (S) IFNλ4 proteins were detected by IFNλ4-specific western blotting (upper panel). Data are representative of n=2 independent experiments. **(e)**Total cellular RNA extracted from the same cells was analyzed for the expression of the ER stress genes *sXBP1* and *CHOP* by RT-qPCR as described in Fig. 4d. Results are expressed as mean±SEM of n=2 independent experiments. Filled circles, IFNλ4; empty triangles, IFNλ4 p131-GFP.

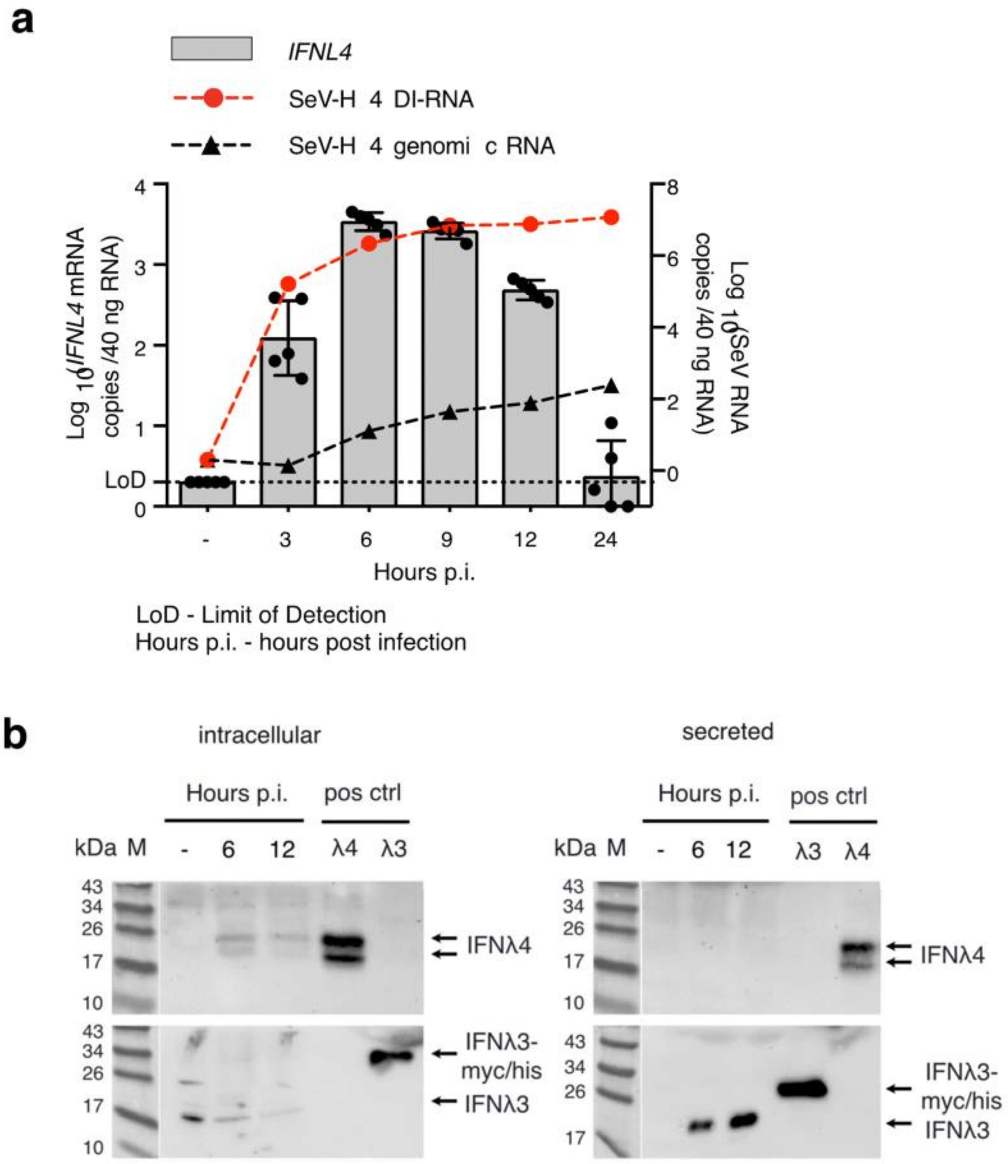

Supplementary Figure 6. IFNλ4 is induced upon viral infection in the lung epithelial cells A549. A549 cells were infected with the Sendai virus H4 strain at a MOI=5 and the infected cells and supernatants were harvested at the indicated time points post-inoculation (hours p.i). **(a)** Kinetics of Sendai virus infection and *IFNL4* mRNA expression. *IFNL4* mRNA (bars), SeV defective interfering RNA (SeV-H4 DI-RNA, red) and SeV genomic RNA (SeV-H4 genomic RNA, black) were quantified in total cellular RNA by RT-qPCR. (-), uninfected control. *IFNL4* mRNA results are expressed as mean±SEM of 2 independent experiments with total of n=5 biological replicates. SeV RNA results are expressed as the mean of n=2 biological replicates of one experiment. Dotted line, lower limit of detection (LoD); negative datapoints and those below LoD are displayed at LoD. **(b)** Intracellular and secreted IFNλ3 and IFNλ4 protein expression was analyzed by western blotting using anti-IFNλ3 and anti-IFNλ4

antibodies. A cell lysate from transfected Huh7 cells were used as a positive control for IFNλ4 detection. Supernatant of Huh7 cells transfected with an IFNλ3-Myc-His expressing vector was used as a positive control for IFNλ3 detection. Data are representative of n=2 independent experiment.

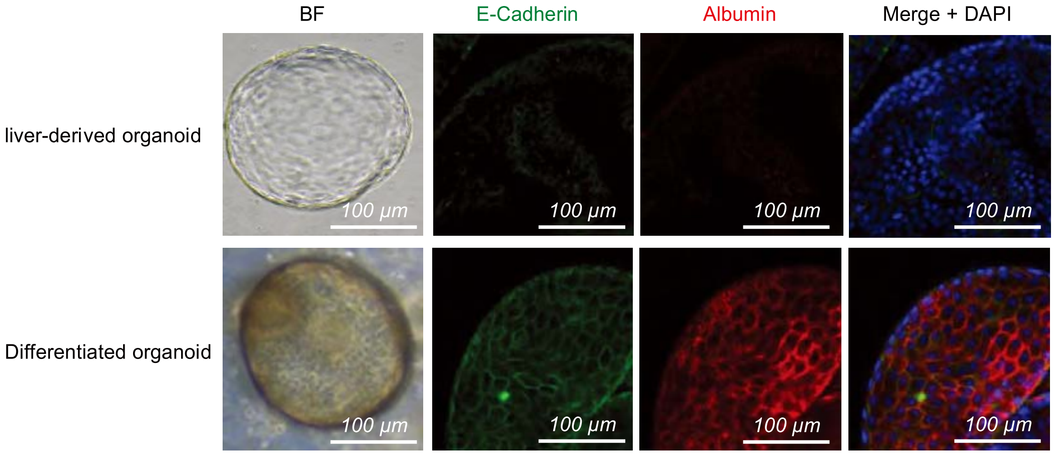

Supplementary Figure 7. Establishment and hepatocyte differentiation of liver-derived organoids. The upper panel shows a representative organoid of a liver-derived organoid culture generated from a liver derived needle biopsy as described in Material and Methods. The liver-derived organoid culture was subjected to hepatocyte differentiation as described in Methods to obtain a differentiated organoid (bottom panel). Organoids are shown in bright field (BF) and were stained by immunofluorescence for the mature hepatocyte markers E-cadherin (green) and Albumin (red) and nuclei were stained by DAPI (blue). Images are representative of n=5 independent experiments. Scale bars are 100 µm.

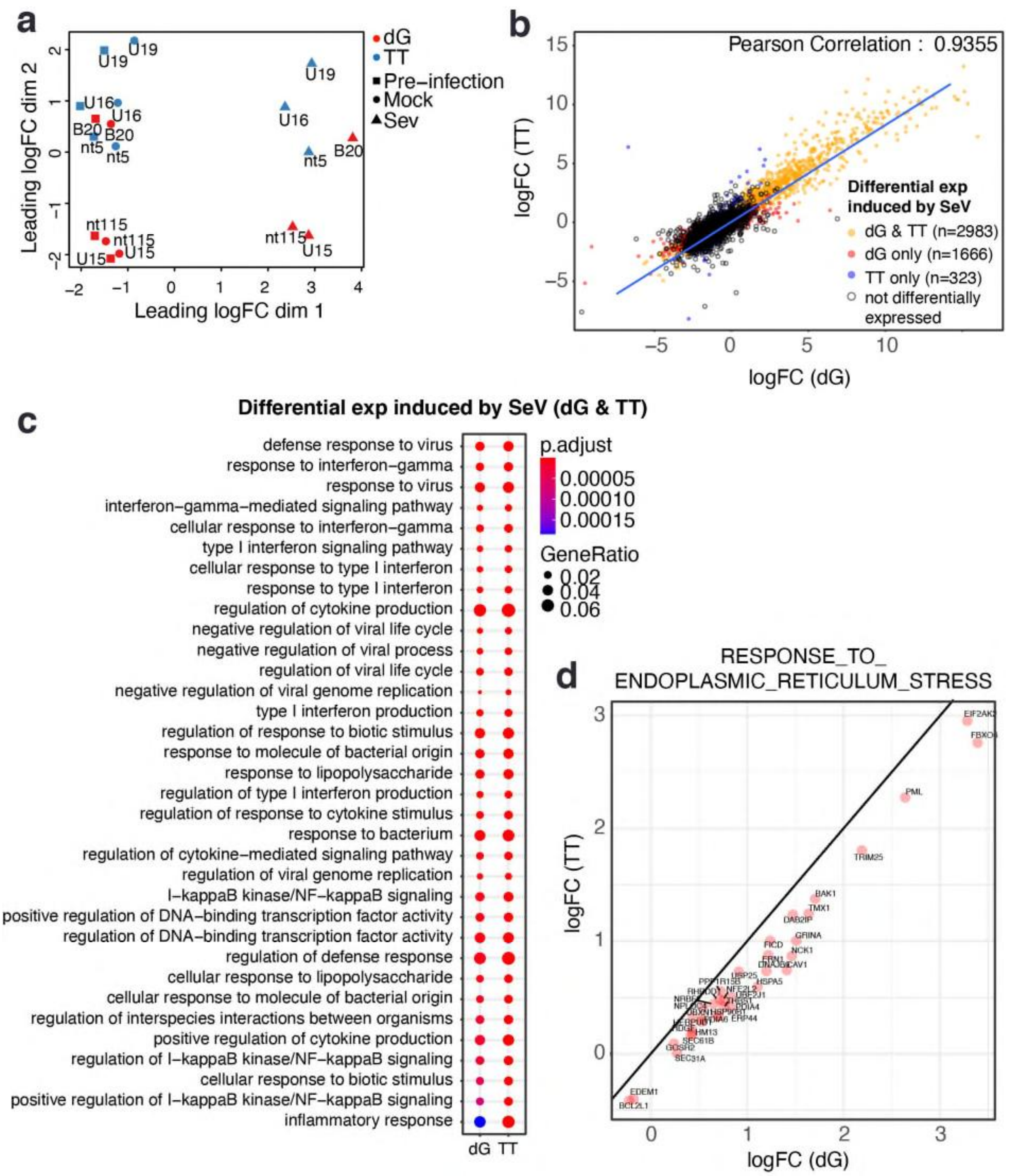

Supplementary Figure 8. Impact of IFNλ4 genotype on global gene expression. Three organoid cultures each of the *dG* (B20, nt115, U15) and *TT* (nt5, U16, U19) genotype were infected with Sendai virus H4 (MOI=10) or mock treated (uninfected control). Global gene expression analysis was performed on organoids before infection (pre-infection) and SeV-infected (SeV) and uninfected (Mock) organoids at 12 hours post infection, respectively. **(a)** Multidimensional scaling (MDS) plot of the transcriptomics profiles using the top 500 (pairwise variable) genes. Colors of the dots

indicate the IFNλ4 genotypes while the shapes indicate SeV infection status. **(b)** Scatter plot shows the log2 fold-change in expression levels induced by SeV infection in organoids of the *TT* genotype (y-axis) against the *dG* genotype (x-axis). Genes with FDR less than 0.05 from each group were considered as differentially expressed and plotted in different colors according to the color key. Correlation of the log2 fold-change in expression levels between the genotypes was evaluated by Pearson correlation. Blue diagonal line indicates y=x. **(c)** Overrepresentation analysis (ORA) of the "Biological Process - Gene Ontology" (GO) terms of the differentially expressed genes was performed in dG and TT organoids, respectively (Methods). Differentially expressed genes were compared against all tested genes (after filtering). Benjamini-Hochberg multiple-testing correction was applied. The top 30 pathways are visualized (Methods). **(d)** Leading genes of the selected pathway "GO_RESPONSE_TO_ENDOPLASMIC_RETICULUM_STRESS" from gene set enrichment analysis with 'fgsea' were plotted as the log2 fold-change in expression levels induced by SeV infection in organoids of the *TT* genotype (y-axis) against the *dG* genotype (x-axis).

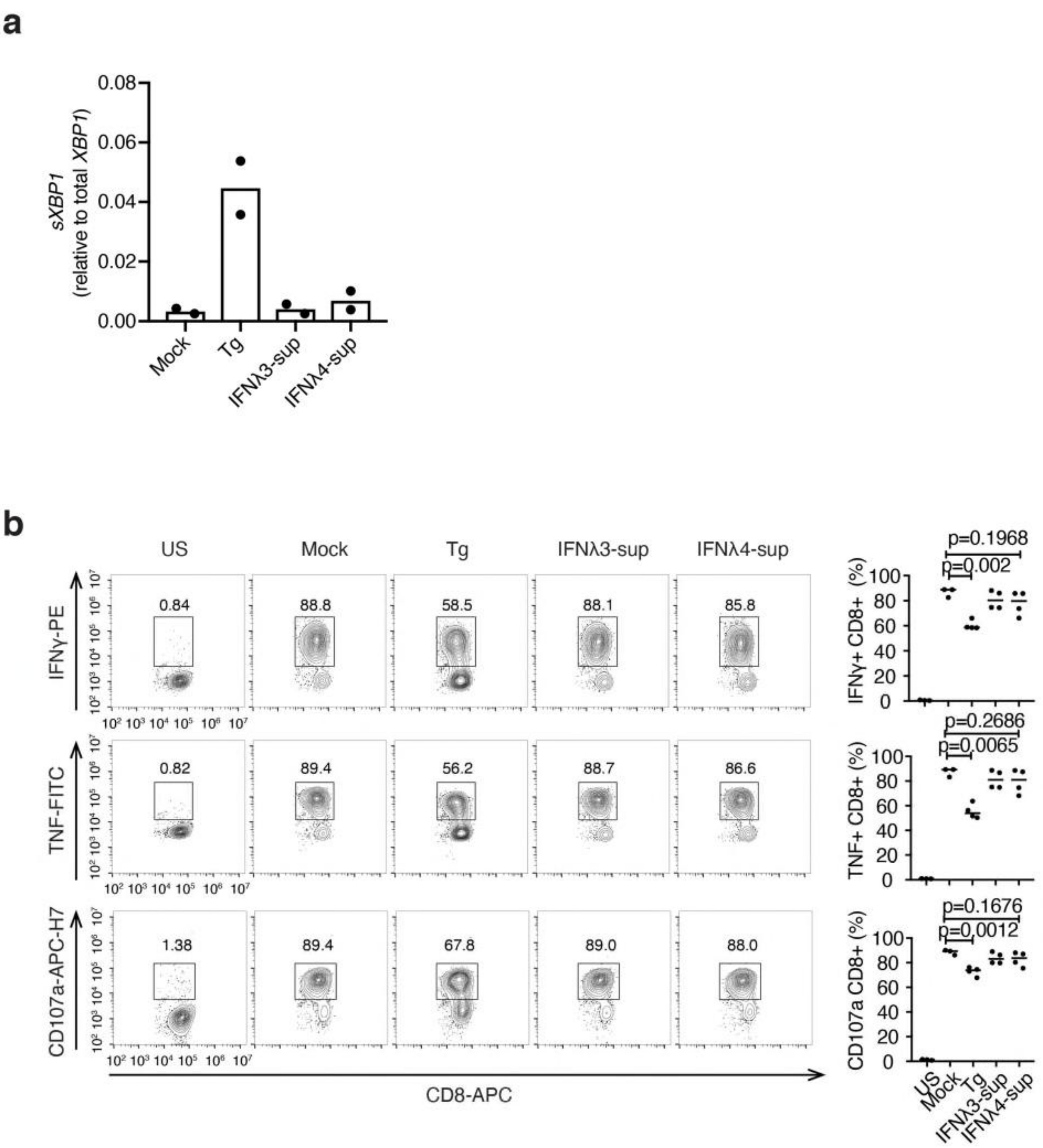

Supplementary Figure 9. Secreted IFNλ4 does not inhibit HCV peptide-specific $CD8^+$ T cell activation. (a,b) Huh7$_{A2}$HCV$_{EM}$ cells were mock treated or incubated with undiluted IFNλ3 or IFNλ4 containing supernatant for 48 hours or stimulated with 2μM Thapsigargin (Tg) for 24 hours. **(a)** Total RNA was extracted from the treated cells and expression of the ER stress marker *sXBP1* was analyzed as described in Fig. 4d. Results are expressed as means of two independent experiments. **(b)** The treated cells were cocultured with the NS5B$_{2594–2602}$-specific $CD8^+$ T cell clone for 5 hours. Flow cytometry analysis was performed exactly as described in Fig. 6a. The right panel shows the results of mean±SEM from n=4 independent experiments. IFNγ+CD8+: p=0.002, mock vs Tg, p=0.1968, mock vs IFNλ4-sup; TNF+CD8+: p=0.0065, mock vs

Tg, p=0.2686, mock vs IFNλ4-sup; CD107a+CD8+: p=0.0012, mock vs Tg, p=0.1676, mock vs IFNλ4-sup, all by two-tailed unpaired t-test.

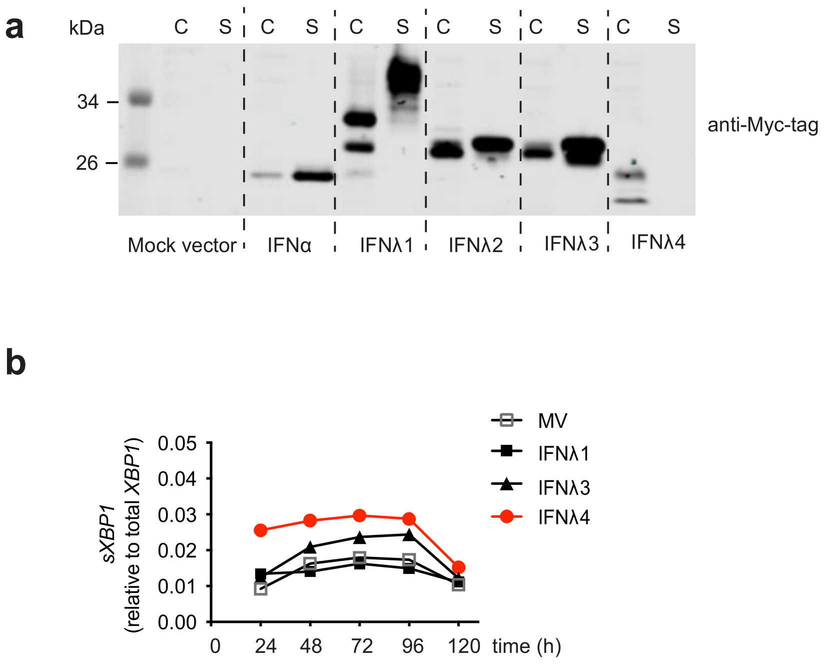

Supplementary Figure 10. IFNλ4 induces ER stress in Huh7$_{A2}$HCV$_{EM}$ cells. (a) Huh7$_{A2}$HCV$_{EM}$ cells were transfected with Myc-His-tagged expression vectors for IFNα, IFNλ1-4 or a mock vector. Cell lysates and supernatants were collected 48 hours after transfection. Intracellular (C) and secreted (S) IFN proteins were detected by Myc-tag-specific western blotting. Data are representative of n=3 independent experiments. **(b)** Total RNA of IFNλ1, IFNλ3, IFNλ4 expression vector and mock vector (MV) transfected Huh7$_{A2}$HCV$_{EM}$ cells was harvested at the indicated time points. Expression of the ER stress marker *sXBP1* was analyzed as described in Fig. 4d. Results are expressed as means of n=2 biological replicates. Empty squares, MV; filled squares, IFNλ1; filled triangles, IFNλ3; red circles, IFNλ4.

a

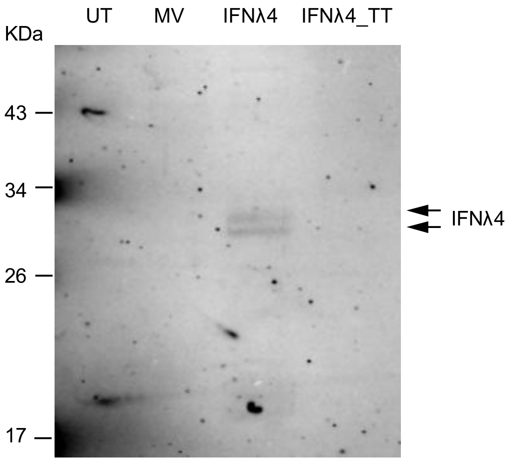

b

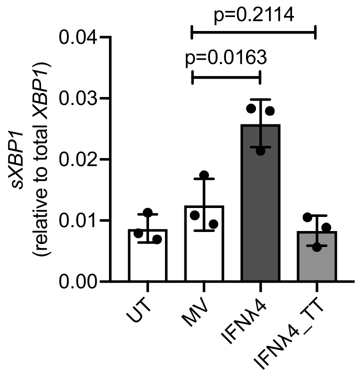

Supplementary Figure 11. IFNλ4-TT does not cause an ER stress response in Huh7 cells. Huh7 cells were transfected with expression vectors encoding IFNλ4, IFNλ4-TT or a mock vector (MV) or were left untreated (UT). **(a)** Cell culture supernatants were collected 24 hours later and subjected to IFNλ4 specific Western blotting. Data are representative of n=3 independent experiments. **(b)** Total RNA was harvested 24 hours after transfection. Expression of the ER stress marker *sXBP1* was analyzed as described in Fig. 4d. Results are expressed as means±SEM of n=3 independent experiments. p=0.0163, MV vs IFNλ4, p=0.2114, MV vs IFNλ4_TT, two-tailed unpaired t-test.

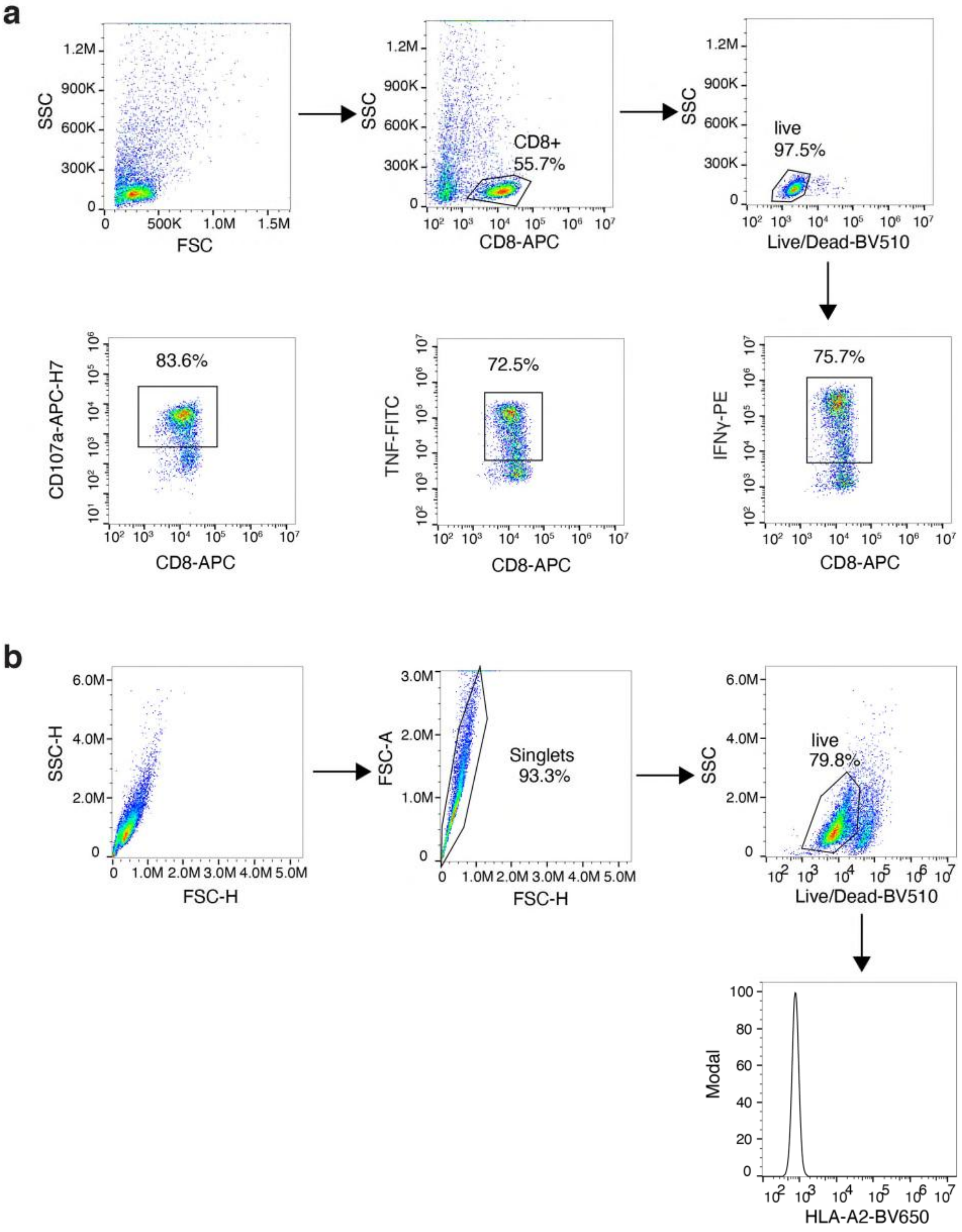

Supplementary Figure 12. Cell gating strategy for flow cytometry analysis. (a) Huh7$_{A2}$HCV$_{EM}$ cells were cocultured with NS5B$_{2594–2602}$-specific CD8^{+} T cells as described in Fig. 6a and 6d. Live CD8^{+} T cells were gated as indicated. The gating for determining the frequency of IFNγ-, TNF- and CD107a-positive CD8^{+} T cells is shown. This gating strategy was applied to the experiments presented in Fig. 6a and 6d, Fig. 7c and Supplementary Fig. 9b. **(b)** Huh7$_{A2}$HCV$_{EM}$ cells were transfected with

expression vectors encoding IFNλ1, IFNλ3, IFNλ4 or a mock vector (MV) and 48 hours later, surface expression of HLA-A2 was analyzed by HLA-A2 BV650-specific flow cytometry. Live single cells were gated as indicated. This gating strategy was used for the experiments presented in Fig. 6b.

Supplementary Table 1: Reagent or resource.

Reagent or resource	Source	Identifier	dilution
**Antibodies**			
Anti-IFNλ4 (rabbit mAb), 1 mg/ml	Abcam	ab196984	1:1000
Anti-IL28B, 1 mg/ml	Abcam	ab125388	1:1000
Anti-Giantin (9B6), 1 mg/ml	Abcam	ab37266	1:1000
Anti-IRE1 (phospho S724), 1 mg/ml	Abcam	ab48187	1:1000
Anti-human CD8-APC	BD Pharmigen	555369	1:50
Anti-human TNFα-FITC, 3.2 µg/ml	BD Pharmigen	340511	1:2
Anti-human CD107a-APC-H7 (H4A3)	BD Pharmigen	561343	1:25
Anti-STAT2, 0.5 mg/ml	BD Transduction Lab	S21220/610188	1:500
Anti-Bip/GRP78, 250 µg/ml	BD Transduction Lab	610978	1:1000
Anti-human HLA-A2-BV650 (BB7.2), 100 µg/ml	BioLegend	343323	1:50
Anti-human IFNγ-PE (4S.B3), 200 µg/ml	BioLegend	502510	1:20
Anti-ATF4 (D4B8)	Cell Signaling	11815	1:1000
Anti-ATF6 (D4Z8V)	Cell Signaling	65880	1:1000
Anti-BSA (D1C8Q)	Cell Signaling	23053	1:2000
Anti-IRE1α (14C10)	Cell Signaling	3294	1:1000
Anti-Myc-Tag (71D10)	Cell Signaling	2278	1:1000
Anti-phospho-STAT1 (Tyr701) (58D6)	Cell Signaling	9167	1:1000
Anti-STAT1 (9H2)	Cell Signaling	9176	1:1000
Anti-phospho-eIF2α (Ser51)	Cell Signaling	9721	1:1000
Anti-eIF2α (L57A5)	Cell Signaling	2103	1:500

Supplementary Table 1: Reagent or resource (continued).

Anti-E Cadherin (24E10)	Cell Signaling	3195	1:1000
Anti-PERK (C33E10)	Cell Signaling	3192	1:1000
Anti-IFNλ4 (rabbit pAb), 0.6 mg/ml	Icosagen	600-100	1:600
IRDye 680RD anti-mouse, 1 mg/ml	Licor	926-68070	1:10000
IRDye 800CW anti-rabbit, 1 mg/ml	Licor	926-32211	1:10000
Anti-IFNλ4 (mouse mAb), 1 mg/ml	Merck Millipore	MABF227	1:1000
Anti-phospo-STAT2 (Tyr689)	Merck Millipore	07-224	1:500
Anti-Calnexin (AF18), 50 µg/ml	Santa-Cruz	sc-23954	1:500
Anti-FLAG M2, 1 mg/ml	SigmaAldrich	F1804	1:1000
Anti-β actin, clone AC-15	SigmaAldrich	A5441	1:10000
Anti-phospho-PERK (Thr981), 1 mg/ml	ThermoFisher Scientific	PA5-40294	1:500
Alexa Fluor 647 goat anti-rabbit IgG, 2 mg/ml	ThermoFisher Scientific	A21244	1:1000
Fluorescein goat anti-mouse IgG, 2 mg/ml	ThermoFisher Scientific	F2761	1:400
Goat anti-Rabbit IgG (H+L) Secondary Antibody, HRP, 1mg/ml	ThermoFisher Scientific	31466	1:5000
**Chemicals, cytokines and peptides**			
Trizol	Ambion	15596018	
BME2	Amsbio		
BMP7	Amsbio		
Brefeldin A	eBioscience	450651	
HCV NS5B (2594-2602) HLA-A*02:01 ALYDVVTKL	Genaxxon Bioscience	P2279.9505	
Blasticidin S hydrochloride	Gibco	R21001	
Expand High Fidelity PCR System	Roche	11732641001	

Supplementary Table 1: Reagent or resource (continued).

L-Glutamine	Gibco	25030081	
G418	Merck Calbiochem	345810	
NucleoSpin Gel and PCR clean-up	Macherey-Nagel	740611.250	
Protein deglycosylation mix	New England BioLabs	P6039S	
PNGase F	New England BioLabs	P0705S	
O-Glycosidase	New England BioLabs	P0733S	
β-N-Acetyl glucosaminidase	New England BioLabs	P0732S	
β1-4 Galactosidase	New England BioLabs	P0730S	
α 2-3,6,8 Neuraminidase	New England BioLabs	P0720S	
Polyethylenimine	Polysciences	23966	
Recombinant IFNa (Roferon-A)	Roche Pharma	Medical product	
Recombinant Viral B18R Protein	R&D Systems	8185-BR	
Collagenase IV	SigmaAldrich	C5138	
Deoxyribonuclease I	SigmaAldrich	DN25	
Phytohemagglutinin-M (PHA-M)	SigmaAldrich	11082132001	
cOmplete Proteinase Inhibitor Cocktail	SigmaAldrich	11697498001	
Thapsigargin	SigmaAldrich	T9033	
OptiPrep	STEMCELL Technologies	07820	
recombinant human IL-2	STEMCELL Technologies	78036	
Penicillin/Streptomycin	ThermoFisher Scientific	15070063	

Supplementary Table 2: Primers for cloning.

Gene	Forward primer	Reverse primer
IFNλ1	CTGGCAGCAAATGATATCGGTACCATGCCGCTGGGGAAGCA	CTTGTAATCACTTCCGGTGGACTCAGGGTGGGT
IFNλ2	CTGGCAGCAAATGATATCGGTACCATGAAACTAGACATGACT	CTTGTAATCACTTCCGACACACAGGTCCCCACT
IFNλ3	CTGGCAGCAAATGATATCGGTACCATGACCGGGGACTG	CTTGTAATCCAGGATATCTCACTTGTCATCGTCATCCTTGTAA
IFNλ4	CTGGCAGCAAATGATATCGGTACCATGCGGCCGAGTGTCT	CTTGTAATCCAGGATATCTCACTTGTCATCGTCATCCTTGTAA
IFNα	CTGGCAGCAAATGATATCGGTACCATGGCCTCGCCCT	CTTGTAATCCAGGATATCTCACTTGTCATCGTCATCCTTGTAA

Supplementary Table 3: Primers for quantitative RT-qPCR.

Gene	Forward primer	Reverse primer
IFNL1	CACAGGAGCTAGCGAGCTTCA	TTTTCAGCTTGAGTGACTCTTCCA
IFNL2/3	GCCAAAGATGCCTTAGAAGAG	CAGAACCTTCAGCGTCAGG
IFNB	AGTAGGCGACACTGTTCGTG	AGCCTCCCATTCAATTGCCA
ISG15	TCCTGCTGGTGGTGGACAA	TTGTTATTCCTCACCAGGATGCT
Mx1	GTGCATTGCAGAAGGTCAGA	TCAGGAGCCAGCTGTAGGTGT
IRF1	GCGACCGCCGAATCG	TGGTTGGAATTAATCTGCATCTCTAG
HCV RNA	TCTGCGGAACCGGTGAGTA	TCAGGCAGTACCACAAGGC
SeV-H4 DI	TTTCCTATCGAGGCACCCCA	TTCCCGAGTAACCCATTCGTG
SeV-H4 genomic	GGATCACTAGGTGATATCGAGC	ACCAGACAAGAGTTTAAGAGATATGTATC
sXBP1	CTGAGTCCGAATCAGGTGCAG	ATCCATGGGGAGATGTTCTGG
Total XBP1	TGGCCGGGTCTGCTGAGTCCG	ATCCATGGGGAGATGTTCTGG
CHOP	AGAACCAGGAAACGGAAACAGA	TCTCCTTCATGCGCTGCTTT
GAPDH	AGGTGAAGGTCGGAGTCAACG	TGGAAGATGGTGATGGGATTTC

Supplementary Table 4: List of liver-derived organoids.

Organoid line	rs368234815 (+/- IFNλ4)	rs117648444 (IFNλ4 P/S70)	Disease background	RNAseq
B16	*ΔG/ ΔG*	*C/C*	HBV	no
U12	*ΔG/TT*	*C/T*	Histo normal	no
B13	*TT/TT*	*C/C*	HBV	no
B20	*ΔG/TT*	*C/T*	HBV, NASH	yes
nt115	*ΔG/ ΔG*	*T/T*	NASH	yes
U15	*ΔG/ ΔG*	*C/C*	ALD, cirrhosis	yes
nt5	*TT/TT*	*C/C*	ALD, cirrhosis	yes
U16	*TT/TT*	*n.a.*	Other	yes
U19	*TT/TT*	*C/C*	ALD	yes

Supplementary Methods

```
library(edgeR)
library(limma)
library(RColorBrewer)
library(pheatmap)
library(ggplot2)
library(ggrepel)
library(data.table)
library(fgsea)
library(dplyr)
library(conflicted)
conflict_prefer("select", "dplyr")
conflict_prefer("paste", "base")
library(clusterProfiler)
library(org.Hs.eg.db)
library(AnnotationDbi)
library(grid)

IFNL4.all.counts.no_U20<-read.csv("all.genes.expected_count.results_no
U20-2.csv")
IFNL4.all.counts.no_U20 <- subset(IFNL4.all.counts.no_U20,
gene_biotype=="protein_coding")

IFNL4.no_U20.DGE<- DGEList(counts=IFNL4.all.counts.no_U20[,4:21],
genes=IFNL4.all.counts.no_U20[,1:3])
keep <- rowSums(cpm(IFNL4.no_U20.DGE)>1) >= 3
IFNL4.no_U20.DGE.filter <- IFNL4.no_U20.DGE[keep, , keep.lib.sizes=FALSE]
IFNL4.no_U20.DGE.filter <- calcNormFactors(IFNL4.no_U20.DGE.filter)

cols<-brewer.pal(n=3,name="Set1")
col2<-cols[x2$IFNL4genotype]
pch.list_2<-c(15,16,17)[x2$Treatment]
par(xpd=TRUE, mar = par()$mar + c(0,0,0,6))
IFNL4.all.counts.no_U20.MDS=plotMDS(IFNL4.no_U20.DGE.filter,col=col2, pch
=pch.list_2, cex = 3)
text(IFNL4.all.counts.no_U20.MDS,labels=x2$OrgLine, cex=1.2, pos=1)
legend(4.2,2,col=cols,legend=levels(x2$IFNL4genotype), pch = 16, cex = 1,
bty = "n",y.intersp = 0.5)
legend(4.2, 1.5,legend=c("Pre-infection", "Mock", "Sev"), pch =
pch.list_2, cex = 1, bty = "n",y.intersp = 0.5)
par(mar=c(5, 4, 4, 2) + 0.1)

IFNL4genotype<-factor(rep(c("dG","TT"),times=c(9,9)))
Treatment<-factor(rep(c("PM","PM","S"),length.out=18))
OrgLine<-
factor(rep(c("B20","nt115","U15","nt5","U16","U19"),times=c(3,3,3,3,3,3))
, levels = c("B20","nt115","U15","nt5","U16","U19"))
x2<-data.frame(Sample=colnames(IFNL4.no_U20.DGE.filter),IFNL4genotype,
Treatment, OrgLine)
x2

Group2 <- factor(paste(x2$IFNL4genotype,x2$Treatment,sep="."))
cbind(x2,Group2=Group2)

design <- model.matrix(~0+Group2+as.numeric(x2$OrgLine))
rownames(design) <- colnames(IFNL4.no_U20.DGE.filter)
```

```
colnames(design)[1:4] <- levels(Group2)
colnames(design)[5] <- "OrgLine"
colnames(design)
design

my.contrasts <- makeContrasts(SvsPM.dG=dG.S-dG.PM,
                              SvsPM.TT=TT.S-TT.PM,
                              levels = design)
IFNL4.no_U20.DGE.filter<- estimateDisp(IFNL4.no_U20.DGE.filter, design,
robust=TRUE)
IFNL4.no_U20.DGE.filter$common.dispersion
plotBCV(IFNL4.no_U20.DGE.filter)

IFNL4.no_U20.DGE.filter.fit <- glmQLFit(IFNL4.no_U20.DGE.filter, design)
plotQLDisp(IFNL4.no_U20.DGE.filter.fit)

qlf.SvsPM.dG<-glmQLFTest(IFNL4.no_U20.DGE.filter.fit,
contrast=my.contrasts[,"SvsPM.dG"])
summary(decideTests(qlf.SvsPM.dG))
topTags(qlf.SvsPM.dG)
t1<-topTags(qlf.SvsPM.dG,n=4649)
t1_all<-topTags(qlf.SvsPM.dG,n=13147)
t1_all<-t1_all$table

qlf.SvsPM.TT<-glmQLFTest(IFNL4.no_U20.DGE.filter.fit,
contrast=my.contrasts[,"SvsPM.TT"])
summary(decideTests(qlf.SvsPM.TT))
topTags(qlf.SvsPM.TT)
t2<-topTags(qlf.SvsPM.TT,n=3306)
t2_all<-topTags(qlf.SvsPM.TT,n=13147)
t2_all<-t2_all$table[match(t1_all$gene_id,t2_all$table$gene_id),]

df<-cbind(t1_all,t2_all$logFC,t2_all$FDR)
colnames(df)[c(4,8:10)]<-c("logFC_dG","FDR_dG","logFC_TT","FDR_TT")
df$threshold<-
ifelse(df$FDR_dG<0.05&df$FDR_TT<0.05,"both",ifelse(df$FDR_dG<0.05,"dG",if
else(df$FDR_TT<0.05,"TT","not_DE")))
cols<-c("both"="orange","dG"="red","TT"="blue","not_DE"="black")
Pearson_correlation<-cor(df$logFC_dG,df$logFC_TT)
library(grid)
grob= grobTree(textGrob(paste("Pearson Correlation : ",
round(cor(df$logFC_dG,df$logFC_TT), 4) ),
                        x = 0.63, y = 0.97, hjust = 0, gp = gpar(col =
"black")))


ggplot(data=df,
       aes(x=logFC_dG, y =logFC_TT))+
  geom_point(aes(shape=threshold, color = threshold),
alpha=0.3,size=2.5)+
  scale_shape_manual(values=c(16, 16, 1,16))+
  scale_colour_manual(values = cols)+
  xlim(c(-8, 15)) +
  ylim(c(-8, 15)) +
  xlab("dG_logFC_(S/PM)") + ylab("TT_logFC_(S/PM)") +
  theme_bw() +
  theme(legend.position="right",panel.background = element_blank())+
  annotation_custom(grob)+
```

```
  geom_smooth(method=lm)


go_enrich_dG <- enrichGO(gene = t1[[1]]$gene_id,
                         universe =
IFNL4.no_U20.DGE.filter$genes$gene_id,
                         OrgDb = org.Hs.eg.db,
                         keyType = 'ENSEMBL',
                         ont = "BP",
                         pvalueCutoff = 0.05,
                         qvalueCutoff = 0.2)
write.csv(as.data.frame(go_enrich_dG)[,-8], file="gobp_ora_dG_gobp.csv",
quote=FALSE, row.names=FALSE)
pdf("gobp_ora_dG_gobp.pdf")
dotplot(go_enrich_dG, showCategory=20)
dev.off()

go_enrich_TT <- enrichGO(gene = t2[[1]]$gene_id,
                         universe =
IFNL4.no_U20.DGE.filter$genes$gene_id,
                         OrgDb = org.Hs.eg.db,
                         keyType = 'ENSEMBL',
                         ont = "BP",
                         pvalueCutoff = 0.05,
                         qvalueCutoff = 0.2)
write.csv(as.data.frame(go_enrich_TT)[,-8], file="gobp_ora_TT_gobp.csv",
quote=FALSE, row.names=FALSE)
pdf("gobp_ora_TT_gobp.pdf")
dotplot(go_enrich_TT, showCategory=20)
dev.off()

m<-merge_result(list("dG"=go_enrich_dG, "TT"=go_enrich_TT))
pdf("gobp_ora_dG&TT_gobp.pdf", width=7, height=12)
dotplot(m, showCategory=30)
dev.off()

generate_gs<-function(df, gmt, id="gene_id") {
  transform_gsea<-function(scores, gmt) {
    scores <- scores[!is.na(scores)]
    gsea<-fgsea(gmt, scores, nperm=10000, minSize = 5)
    gsea$primary<-FALSE
    gsea[which(gsea$pathway %in% collapsePathways(gsea, gmt,
scores)$mainPathways),"primary"]<-TRUE
    return(gsea)
  }

  scores<-df$score
  names(scores)<-df[[id]]

  res_gsea<-transform_gsea(scores, gmt)
  return(list("fgsea"=res_gsea,"pathways"=gmt,"scores"=scores))
}

scores_SvsPM.dG<-data.frame("gene_id"=qlf.SvsPM.dG$gene$gene_id,
"gene_name"=qlf.SvsPM.dG$gene$gene_name,
"score"=sign(qlf.SvsPM.dG$table$logFC)*qlf.SvsPM.dG$table$F)
gobp_gsea_SvsPM.dG<-generate_gs(scores_SvsPM.dG,
gmtPathways("c5.go.bp.v7.2.symbols.gmt"), id="gene_name")
```

```
write.csv(gobp_gsea_SvsPM.dG$fgsea[,-c("leadingEdge")],
file="gobp_gsea_SvsPM.dG.csv", quote=FALSE, row.names=FALSE)

pdf("gobp_gsea_SvsPM.dG.pdf", width=12, height=12)
plotGseaTable(gobp_gsea_SvsPM.dG$pathways[gobp_gsea_SvsPM.dG$fgsea[head(o
rder(pval), n=15)][order(-NES), pathway]], gobp_gsea_SvsPM.dG$scores,
gobp_gsea_SvsPM.dG$fgsea, gseaParam=0.5)
dev.off()

scores_SvsPM.TT<-data.frame("gene_id"=qlf.SvsPM.TT$gene$gene_id,
"gene_name"=qlf.SvsPM.TT$gene$gene_name,
"score"=sign(qlf.SvsPM.TT$table$logFC)*qlf.SvsPM.TT$table$F)
gobp_gsea_SvsPM.TT<-generate_gs(scores_SvsPM.TT,
gmtPathways("c5.go.bp.v7.2.symbols.gmt"), id="gene_name")
write.csv(gobp_gsea_SvsPM.TT$fgsea[,-c("leadingEdge")],
file="gobp_gsea_SvsPM.TT.csv", quote=FALSE, row.names=FALSE)

pdf("gobp_gsea_SvsPM.TT.pdf", width=12, height=12)
plotGseaTable(gobp_gsea_SvsPM.TT$pathways[gobp_gsea_SvsPM.TT$fgsea[head(o
rder(pval), n=15)][order(-NES), pathway]], gobp_gsea_SvsPM.TT$scores,
gobp_gsea_SvsPM.TT$fgsea, gseaParam=0.5)
dev.off()

scores_dGvsTT.S_PM<-data.frame("gene_id"=qlf.dGvsTT.S_PM$gene$gene_id,
"gene_name"=qlf.dGvsTT.S_PM$gene$gene_name,
"score"=sign(qlf.dGvsTT.S_PM$table$logFC)*qlf.dGvsTT.S_PM$table$F)
gobp_gsea_dGvsTT.S_PM<-generate_gs(scores_dGvsTT.S_PM,
gmtPathways("c5.go.bp.v7.2.symbols.gmt"), id="gene_name")
write.csv(gobp_gsea_dGvsTT.S_PM$fgsea[,-c("leadingEdge")],
file="gobp_gsea_dGvsTT.S_PM.csv", quote=FALSE, row.names=FALSE)

pdf("gobp_gsea_dGvsTT.S_PM.pdf", width=12, height=12)
plotGseaTable(gobp_gsea_dGvsTT.S_PM$pathways[gobp_gsea_dGvsTT.S_PM$fgsea[
head(order(pval), n=15)][order(-NES), pathway]],
gobp_gsea_dGvsTT.S_PM$scores, gobp_gsea_dGvsTT.S_PM$fgsea, gseaParam=0.5)
dev.off()

pe_scores<-scores_dGvsTT.S_PM$score
names(pe_scores)<-scores_dGvsTT.S_PM$gene_name

pdf(paste("GO_RESPONSE_TO_ENDOPLASMIC_RETICULUM_STRESS","pdf",sep="."))
plotEnrichment(gmtPathways("c5.go.bp.v7.2.symbols.gmt")[["GO_RESPONSE_TO_
ENDOPLASMIC_RETICULUM_STRESS"]], pe_scores)
dev.off()

df<-cbind(qlf.SvsPM.dG$genes,
qlf.SvsPM.dG$table$logFC,qlf.SvsPM.TT$table$logFC)
colnames(df)[4:5]<-c("lfc_dG","lfc_TT")

df$gset<-"others"
df[which(df$gene_name %in% subset(gobp_gsea_dGvsTT.S_PM$fgsea,
pathway=="GO_RESPONSE_TO_ENDOPLASMIC_RETICULUM_STRESS")$leadingEdge[[1]])
,"gset"]<-"GO_RESPONSE_TO_ENDOPLASMIC_RETICULUM_STRESS"
cols<-
c("GO_RESPONSE_TO_ENDOPLASMIC_RETICULUM_STRESS"="red","others"="darkgrey"
)

gsetplot<-ggplot(data=subset(df,gset!="others"),
```

```
                 aes(x=lfc_dG, y =lfc_TT))+
  geom_point(aes(color = gset),alpha=0.3, size=2.5)+
  scale_colour_manual(values = cols)+
  # xlim(c(-8, 15)) +
  # ylim(c(-8, 15)) +
  xlab("lfc_dG") + ylab("lfc_TT") +
  theme_bw(base_size = 12) +
  theme(legend.position="bottom")+
  geom_label_repel(
    data = subset(df,gset!="others"),
    aes(label = gene_name),
    size = 3,
    box.padding = unit(0.3, "lines"),
    point.padding = unit(0.3, "lines"))+
  geom_abline()

ggsave(filename="GO_RESPONSE_TO_ENDOPLASMIC_RETICULUM_STRESS_SP.pdf",
gsetplot)
```
