# Supplementary material for: Interferon lambda 4 impairs hepatitis C viral antigen presentation and attenuates T cell responses
Source: Nat Commun. 2021 Aug 12;12:4882. doi: 10.1038/s41467-021-25218-x (PMC8360984; doi:10.1038/s41467-021-25218-x)
Supplement: Supplementary file 3 — Description of Additional Supplementary Files [file 41467_2021_25218_MOESM3_ESM.pdf]

Description of Additional Supplementary Files

File Name: Supplementary Data 1
Description: Enriched pathways in the gene set regulated by SeV infection in organoids of dG genotype

File Name: Supplementary Data 2
Description: Enriched pathways in the gene set regulated by SeV infection in organoids of TT genotype
