# Supplementary material for: Interferon lambda 4 impairs hepatitis C viral antigen presentation and attenuates T cell responses
Source: Nat Commun. 2021 Aug 12;12:4882. doi: 10.1038/s41467-021-25218-x (PMC8360984; doi:10.1038/s41467-021-25218-x)
Supplement: Supplementary file 6 — Reporting Summary [file 41467_2021_25218_MOESM6_ESM.pdf]

nature research

Corresponding author(s): Markus H. Heim

Last updated by author(s): Jun 9, 2021

Reporting Summary

Nature Research wishes to improve the reproducibility of the work that we publish. This form provides structure for consistency and transparency in reporting. For further information on Nature Research policies, see our Editorial Policies and the Editorial Policy Checklist.

Statistics

For all statistical analyses, confirm that the following items are present in the figure legend, table legend, main text, or Methods section.

n/a	Confirmed	
[ ]	[x]	The exact sample size (*n*) for each experimental group/condition, given as a discrete number and unit of measurement
[ ]	[x]	A statement on whether measurements were taken from distinct samples or whether the same sample was measured repeatedly
[ ]	[x]	The statistical test(s) used AND whether they are one- or two-sided<br>*Only common tests should be described solely by name; describe more complex techniques in the Methods section.*
[ ]	[x]	A description of all covariates tested
[ ]	[x]	A description of any assumptions or corrections, such as tests of normality and adjustment for multiple comparisons
[ ]	[x]	A full description of the statistical parameters including central tendency (e.g. means) or other basic estimates (e.g. regression coefficient) AND variation (e.g. standard deviation) or associated estimates of uncertainty (e.g. confidence intervals)
[ ]	[x]	For null hypothesis testing, the test statistic (e.g. *F*, *t*, *r*) with confidence intervals, effect sizes, degrees of freedom and *P* value noted<br>*Give P values as exact values whenever suitable.*
[x]	[ ]	For Bayesian analysis, information on the choice of priors and Markov chain Monte Carlo settings
[x]	[ ]	For hierarchical and complex designs, identification of the appropriate level for tests and full reporting of outcomes
[ ]	[x]	Estimates of effect sizes (e.g. Cohen's *d*, Pearson's *r*), indicating how they were calculated

Our web collection on statistics for biologists contains articles on many of the points above.

Software and code

Policy information about availability of computer code

Data collection	Western blot:Odyssey-CLx image system (Licor) and Gel Doc XR+ System (Bio-Rad)<br>qPCR:ABI 7500 or 7500 Fast Real Time PCR System (Applied BiosystemsTM)<br>ELISA: multi-mode microplate reader (Centro XS3 LB960, Berthold Technologies) and Synergy H1 Hybrid Reader (BioTek)<br>Immunofluorescence microscopy: Zeiss LSM 710 confocal microscope (Carl Zeiss)<br>Flow cytometry: CytoFLEX (Beckman)<br>RNA sequencing: NextSeq 550
Data analysis	Western blot: Fiji distribution of ImageJ (version 2.0.0) and Prism8 (GraphPad Software Inc, version 8.4.3)<br>qPCR and ELISA: Prism8 (GraphPad Software Inc, version 8.4.3)<br>Immunofluorescence microscopy: Fiji distribution of ImageJ (version 2.0.0)<br>Flow cytometry: Flowjo 10 .4 (FlowJo, LLC)<br>RNAseq analysis: RTA version 1.18.66.3, RStudio v3.6, STAR, edgeR R package, R-package "fgsea" (version 1.14.0), MSigDB (version 7.2)

For manuscripts utilizing custom algorithms or software that are central to the research but not yet described in published literature, software must be made available to editors and reviewers. We strongly encourage code deposition in a community repository (e.g. GitHub). See the Nature Research guidelines for submitting code & software for further information.

Data

Policy information about availability of data

All manuscripts must include a data availability statement. This statement should provide the following information, where applicable:

- Accession codes, unique identifiers, or web links for publicly available datasets
- A list of figures that have associated raw data
- A description of any restrictions on data availability

All data that support the findings of this study are available within the manuscript and the supplementary information files. The RNA-Seq data reported here has been deposited in https://ega-archive.org/submitter-portal/#/login under accession number: EGAS00001005396. All other primary data is provided in the source data file accompanying this paper.

Field-specific reporting

Please select the one below that is the best fit for your research. If you are not sure, read the appropriate sections before making your selection.

☒ Life sciences ☐ Behavioural & social sciences ☐ Ecological, evolutionary & environmental sciences

For a reference copy of the document with all sections, see nature.com/documents/nr-reporting-summary-flat.pdf

Life sciences study design

All studies must disclose on these points even when the disclosure is negative.

Sample size	No statistical methods were used to predetermine the sample size. The sample size was based on previously published results (PMID: 28900038, 33158978), past experience, pilot experiments, as well as feasibility of experiments (for example, availability of growing liver-derived organoids). Data variance was low in all experiments using cell lines that sample sizes did not constrain analysis.
Data exclusions	No data were excluded.
Replication	Experiments were independently repeated as indicated in the figure legends and could have been successfully replicated. Western blot, gel staining, immunofluorescence and flow cytometry data are representative of 3 or more independent experiments. Rather than biological replicates, we used groups of individual patient derived organoids (B20, nt115, U15,nt5, U16, U19) representing the specific genotype for the experiments presented in Fig. 5C and Supplementary Fig. 8 using organoids.
Randomization	Cell lines used in the study were not randomized. In all organoid experiments, organoids were randomly selected for IFNL4 genotyping and 9 organoids with robust growth of each genotype were finally selected for the experiments reported in Fig. 5 and Supplementary Fig. 8.
Blinding	For experiments using cell lines, the investigators were not blinded during data acquisition and analysis. The application of treatments and processing procedures negated the possibility of blinding but there was no human bias given as all data was analyzed independently using instrumentation. For experiments using organoids, the investigator who grew the organoids was not blinded to the sample allocation. Three other investigators were involved in the following experiments and were not aware of the group allocation during data acquisition.

Reporting for specific materials, systems and methods

We require information from authors about some types of materials, experimental systems and methods used in many studies. Here, indicate whether each material, system or method listed is relevant to your study. If you are not sure if a list item applies to your research, read the appropriate section before selecting a response.

Materials & experimental systems

n/a	Involved in the study	
☐	☒	Antibodies
☐	☒	Eukaryotic cell lines
☒	☐	Palaeontology and archaeology
☒	☐	Animals and other organisms
☐	☒	Human research participants
☒	☐	Clinical data
☒	☐	Dual use research of concern

Methods

n/a	Involved in the study	
☒	☐	ChIP-seq
☐	☒	Flow cytometry
☒	☐	MRI-based neuroimaging

Antibodies

Antibodies used	Anti-IFNλ4 (rabbit mAb), 1 mg/ml, Abcam, ab196984, 1:1000<br>Anti-IL28B, 1 mg/ml ,Abcam, ab125388, 1:1000<br>Anti-Giantin (9B6), 1 mg/ml, Abcam, ab37266, 1:1000

Anti-IRE1 (phospho S724), 1 mg/ml, Abcam, ab48187, 1:1000
Anti-human CD8-APC, BD Pharmigen, 5553689, 1:50
Anti-human TNFα-FITC, 3.2 μg/ml, BD Pharmigen, 340511, 1:2
Anti-human CD107a-APC-H7 (H4A3), BD Pharmigen, 561343, 1:25
Anti-STAT2, 0.5 mg/ml, BD Transduction Lab, S21220/610188, 1:500
Anti-Bip/GRP78, 250 μg/ml, BD Transduction Lab, 610978, 1:1000
Anti-human HLA-A2-BV650 (BB7.2), 100 μg/ml, BioLegend, 343323, 1:50
Anti-human IFNγ-PE (4S.B3), 200 μg/ml, BioLegend, 502510, 1:20
Anti-ATF4 (D4B8), Cell Signaling, 11815, 1:1000
Anti-ATF6 (D4Z8V), Cell Signaling, 65880, 1:1000
Anti-BSA (D1C8Q), Cell Signaling, 23053, 1:2000
Anti-IRE1α (14C10), Cell Signaling, 3294, 1:1000
Anti-Myc-Tag (71D10), Cell Signaling, 2278, 1:1000
Anti-phospho-STAT1 (Tyr701) (58D6), Cell Signaling, 9167, 1:1000
Anti-STAT1 (9H2), Cell Signaling, 9176, 1:1000
Anti-phospho-eIF2α (Ser51), Cell Signaling, 9721, 1:1000
Anti-eIF2α (L57A5), Cell Signaling, 2103, 1:500
Anti-E Cadherin (24E10), Cell Signaling, 3195, 1:1000
Anti-PERK (C33E10), Cell Signaling ,3192, 1:1000
Anti-IFNλ4 (rabbit pAb), 0.6 mg/ml, Icosagen ,600-100, 1:600
IRDye 680RD anti-mouse, 1 mg/ml, Licor, 926-68070, 1:10000
IRDye 800CW anti-rabbit, 1 mg/ml, Licor, 926-32211, 1:10000
Anti-IFNλ4 (mouse mAb), 1 mg/ml ,Merck Millipore, MABF227, 1:1000
Anti-phospo-STAT2 (Tyr689), Merck Millipore, 07-224, 1:500
Anti-Calnexin (AF18), 50 μg/ml, Santa-Cruz, sc-23954, 1:500
Anti-FLAG M2, 1 mg/ml, SigmaAldrich, F1804, 1:1000
Anti-β actin, clone AC-15, SigmaAldrich, A5441, 1:10000
Anti-phospho-PERK (Thr981), 1 mg/ml, ThermoFisher Scientific, PA5-40294, 1:500
Alexa Fluor 647 goat anti-rabbit IgG, 2 mg/ml ThermoFisher Scientific, A21244 1:1000
Fluorescein goat anti-mouse IgG, 2 mg/ml, ThermoFisher Scientific F2761, 1:400
Goat anti-Rabbit IgG (H+L) Secondary Antibody, HRP, 1mg/ml, ThermoFisher Scientific, 31466, 1:5000

Validation

Antibody validations were performed by suppliers. All respective validation data are available on the manufacturer's website. Additional validation has also been given in previous publication with PubMed IDs listed:
Anti-IFNλ4 (rabbit mAb), PMID: 23291588, 26134097
Anti-IL28B, 1 mg/ml, PMID: 26134097
Anti-Giantin (9B6), PMID: 32325029, 31847122
Anti-IRE1 (phospho S724), PMID: 31746423, 31894849
Anti-human CD8-APC, Mouse IgG1, κ, PMID: 31984629
Anti-human TNFα-FITC, Mouse IgG1, PMID: 7684430
Anti-human CD107a-APC-H7 (H4A3), Mouse BALB/c IgG1, κ, PMID: 12377779
Anti-STAT2, 0.5 mg/ml, Mouse IgG2a, PMID: 11704862
Anti-Bip/GRP78, 250 μg/ml, Mouse IgG2a, PMID: 12429840
Anti-human HLA-A2-BV650 (BB7.2), Mouse IgG2b, κ, PMID: 30718505
Anti-human IFNγ-PE (4S.B3), Mouse IgG1, κ, PMID: 29752063
Anti-ATF4 (D4B8), Rabbit IgG, PMID: 33446657
Anti-ATF6 (D4Z8V), Rabbit IgG, PMID: 33303737
Anti-BSA (D1C8Q), Rabbit IgG, manufacture's website: https://www.cellsignal.com/products/primary-antibodies/bsa-d1c8q-rabbit-mab/23053?_=1623244599857&Ntt=23053&tahead=true
Anti-IRE1α (14C10), Rabbit IgG, PMID: 33184288
Anti-Myc-Tag (71D10), Rabbit IgG, PMID: 33479206
Anti-phospho-STAT1 (Tyr701) (58D6), Rabbit IgG, PMID: 33510287
Anti-STAT1 (9H2), Mouse IgG1, PMID: 32049022
Anti-phospho-eIF2α (Ser51), Rabbit, PMID: 33313903
Anti-eIF2α (L57A5), Mouse IgG2b, PMID: 33008000
Anti-E Cadherin (24E10), Rabbit IgG, PMID: 33637728
Anti-PERK (C33E10), Rabbit IgG, PMID: 33168829
Anti-IFNλ4 (rabbit pAb), 0.6 mg/ml, validated in house
Anti-IFNλ4 (mouse mAb), 1 mg/ml, Mouse IgG1κ, PMID: 23291588
Anti-phospo-STAT2 (Tyr689), Rabbit IgG, manufacture's website: https://www.merckmillipore.com/CH/de/product/Anti-phospho-STAT2-Tyr689-Antibody,MM_NF-07-224
Anti-Calnexin (AF18), 50 μg/ml, Mouse IgG1 κ, PMID: 30759394
Anti-FLAG M2, 1 mg/ml, Mouse IgG1, manufacture's website: https://www.sigmaaldrich.com/CH/en/product/sigma/f1804?context=product
Anti-β actin, clone AC-15, Mouse IgG1, manufacture's website: https://www.sigmaaldrich.com/CH/en/product/sigma/a5441?context=product
Anti-phospho-PERK (Thr981), 1 mg/ml, Rabbit IgG, PMID: 31767864

Eukaryotic cell lines

Policy information about cell lines

Cell line source(s)	A549 (ATCC® Number: CCL-185™)<br>Tetracycline-inducible HepG2 cell lines (gifts from Dr. Ludmila Prokunina-Olsson of Division of Cancer Epidemiology & Genetics, NIH. PMID: 23291588, 26134097)<br>Huh7A2HCVEM and Huh7A2HCV replicon cells (generated by Dr. Volker Lohmann)<br>NS5B2594-2602-specific CD8+ T cell (generated by Dr. Robert Thimme)<br>Huh-7 and there derivatives (Huh-7.5 and Huh7.5.1) are available from Apath (http://apath.com)<br>Huh7-LR (generated in house. PMID: 24752298)
Authentication	A549 cells were authenticated using karyotyping. Tetracycline-inducible HepG2 cell lines were validated for expression of IFNλ4 proteins upon adding doxycycline. Huh7A2HCVEM and Huh7A2HCV replicon cells were validated for determing HCV replication levels using the Steady-Glo Luciferase Assay System (Promega) according to the manufacturer's instructions. NS5B2594-2602-specific CD8+ T cells were validated for killing the epitope-matched Huh7A2HCVEM but not the mismatched Huh7A2HCV replicon cells. Huh7-LR cells were validated using RT-qPCR. Huh7 cells were not authenticated.
Mycoplasma contamination	All cell lines were regularly tested negative for mycoplasma contamination.
Commonly misidentified lines<br>(See ICLAC register)	No misidentified lines were used.

Human research participants

Policy information about studies involving human research participants

Population characteristics	Patients enrolled in this study presented with mild unclear hepatopathy and underwent a diagnostic liver biopsy.
Recruitment	There was no selection criteria for establishing organoid cultures from biopsies. Established organoids were genotyped for the IFNL4 polymorphisms and used accordingly.
Ethics oversight	The study was carried out in accordance with The Code of Ethics of the World Medical Association (Declaration of Helsinki) and was approved by the Ethics Committee of North Western Switzerland (Authorization number EKNZ 2014–362). Written informed consent was obtained from all patients enrolled in this study.

Note that full information on the approval of the study protocol must also be provided in the manuscript.

Flow Cytometry

Plots

Confirm that:

- [x] The axis labels state the marker and fluorochrome used (e.g. CD4-FITC).
- [x] The axis scales are clearly visible. Include numbers along axes only for bottom left plot of group (a 'group' is an analysis of identical markers).
- [x] All plots are contour plots with outliers or pseudocolor plots.
- [x] A numerical value for number of cells or percentage (with statistics) is provided.

Methodology

Sample preparation	For data reported in Fig. 6b, 0.5x105 Huh7A2HCVEM replicon cells/well were seeded in a 24-well plate one day before PEI transfection with expression plasmid for IFNλ1, IFNλ3 and IFNλ4 (described above). Cells were harvested 48 hours-post transfection. Live/dead cell staining was perform using Zombi aqua fixable viability kit (Biolegend). After washing with 2% BSA, cells were stained with anti-human HLA-A2 BV650 antibody.<br>For data reported in Fig.6a, Huh7A2HCVEM replicon cells were seeded and transfected as described above. 48 hours-post transfection, replicon cells were co-cultured with NS5B2594-2602-specific CD8+ T cells at an effector-to-target ration (E/T) of 2:1 in a total of 500 µl medium in the presence of brefeldin A. After 5 hours of coculture, cells were harvested. Live/dead cell staining was perform using Zombi aqua fixable viability kit (Biolegend). After washing with 2% BSA, cells were stained with anti-CD8-APC (1:50) and anti-CD107a-APC-H7 (1:25) diluted in 2% BSA for 30 minutes at 4oC. Thereafter, cells were fixed (IC fixation buffer, eBioscienceTM) and permeabilized (permeabilization buffer, eBioscienceTM) according to manufacturer's instruction. Intracellular IFNγ and TNFα staining was performed with anti-IFNγ-PE (1:20) and anti-TNFα-FITC (1:2) diluted in permeabilization buffer for 30 minutes at 4oC.<br>For data reported in Fig.6d, Huh7A2HCV cells were transfected with the IFN expression plasmids as described above. 48 hours-post transfection, the NS5B2594–2602-peptide ALYDVVTKL (10 µg/ml) was added to the cell culture medium for 1 h at 37oC. Then, the cells were washed twice with fresh medium before co-culturing with the CD8+ T cells in the presence of brefeldin A and subsequent flow cytometry analysis of the CD8+ T cells as described above.<br>For data reported in Fig. 7b, Huh7A2HCVEM cells were transfected with expression vectors for IFNλ4 and IFNλ4-TT or a mock

	vector (MV) or were left untreated (UT). 48 hours-post transfection, replicon cells were co-cultured with NS5B2594-2602-specific CD8+ T cells as described in Fig. 6a. Flow cytometry analysis was performed exactly as described in Fig. 6a.<br>For data reported in Supplementary Fig. 9b, Huh7A2HCVEM cells were mock treated or incubated with undiluted IFNλ3 or IFNλ4 containing supernatant for 48 hours or stimulated with 2µM Thapsigargin (Tg) for 24 hours. The treated cells were cocultured with the NS5B2594–2602-specific CD8+ T cell clone for 5 hours. Flow cytometry analysis was performed exactly as described in Fig. 6a.
Instrument	CytoFLEX (Beckman)
Software	Flowjo 10.4 (FlowJo, LLC)
Cell population abundance	No cell sorting was performed.
Gating strategy	The cell population was defined using FSC-H and SSC-H, followed by gating on live cells using Live/dead cell staining.

☒ Tick this box to confirm that a figure exemplifying the gating strategy is provided in the Supplementary Information.
